# Comparing attitudes about legal sanctions and teratogenic effects for cocaine, alcohol, tobacco and caffeine: A randomized, independent samples design

**DOI:** 10.1186/1747-597X-1-4

**Published:** 2006-02-01

**Authors:** Harvey J Ginsburg, Paul Raffeld, Kelly L Alanis, Angela S Boyce

**Affiliations:** 1 Department of Psychology, Texas State University – San Marcos, San Marcos, Texas 78666, USA

## Abstract

**Background:**

Establishing more sensible measures to treat cocaine-addicted mothers and their children is essential for improving U.S. drug policy. Favorable post-natal environments have moderated potential deleterious prenatal effects. However, since cocaine is an illicit substance having long been demonized, we hypothesized that attitudes toward prenatal cocaine exposure would be more negative than for licit substances, alcohol, nicotine and caffeine. Further, media portrayals about long-term outcomes were hypothesized to influence viewers' attitudes, measured immediately post-viewing. Reducing popular crack baby stigmas could influence future policy decisions by legislators.

In Study 1, 336 participants were randomly assigned to 1 of 4 conditions describing hypothetical legal sanction scenarios for pregnant women using cocaine, alcohol, nicotine or caffeine. Participants rated legal sanctions against pregnant women who used one of these substances and risk potential for developing children.

In Study 2, 139 participants were randomly assigned to positive, neutral and negative media conditions. Immediately post-viewing, participants rated prenatal cocaine-exposed or non-exposed teens for their academic performance and risk for problems at age18.

**Results:**

Participants in Study 1 imposed significantly greater legal sanctions for cocaine, perceiving prenatal cocaine exposure as more harmful than alcohol, nicotine or caffeine. A one-way ANOVA for independent samples showed significant differences, beyond .0001. Post-hoc Sheffe test illustrated that cocaine was rated differently from other substances.

In Study 2, a one-way ANOVA for independent samples was performed on difference scores for the positive, neutral or negative media conditions about prenatal cocaine exposure. Participants in the neutral and negative media conditions estimated significantly lower grade point averages and more problems for the teen with prenatal cocaine exposure than for the non-exposed teen beyond .0001 alpha level. The positive media program closed estimated grade point average differences and risks of later problems to a non-statistically significant margin, *p *>.05.

**Conclusion:**

Ratings for prenatal cocaine were more negative than comparable ratings for alcohol, nicotine or caffeine exposure. Stereotypes can be reduced, showing viewers that positive postnatal environments ameliorate potential teratogenic effects of cocaine. Reducing negative stereotypes for crack babies may be a requisite for substantive changes in current policy.

## Background

An essential need in contemporary U.S. drug policy is to establish sensible and sensitive measures to treat cocaine-addicted mothers and their children [1]. The debate about long-term, non-clinical effects of prenatal cocaine exposure has continued for over two decades. Meta-analyses of the long-term effects of prenatal cocaine exposure have shown inconsistent and subtle effects [2]. Relative to comparable non-cocaine exposed infants, cocaine-exposed infants were reported to perform more poorly on visual recognition tasks [3]. This finding for infants is consistent with the findings that left hemisphere visual attention was diminished in young children exposed to cocaine during pregnancy [4]. Prenatal cocaine exposure was also related to task persistence at ages 3, 5 and 7 years when polydrug exposure was co-varied [5]. However, others investigators reported no long-term effects in 3 to 6 year-old children [6], comparing cocaine/polydrug-exposed children to those with no prenatal substance exposure when ethnicity, age, gender and socio-economic status were controlled. Similarly, when the play behaviors of unexposed toddlers and toddlers exposed to cocaine in utero were compared, no differences were reported [7]. Regarding long-term effects of prenatal cocaine exposure, firm conclusions still appear unwarranted.

Although the scientific debate about prenatal cocaine exposure has continued for over two decades, a dearth of information exists for public attitudes toward prenatal cocaine exposure. Results from a study examining performance ratings of children labeled as normal or prenatal cocaine-exposed reflected negative attitudes toward the latter when college undergraduates were shown videotapes of the same children doing identical tasks. Participants rated the cocaine-exposed labeled child lower in task performance and less favorably than ones labeled unexposed [8].

A brief review of the past literature illustrates some of the reasons that public attitudes toward children and adolescents exposed to cocaine during prenatal development may be negative. Investigators initially reported decreased birth weight, increased premature births, sudden infant death syndrome, intrauterine growth retardation, decreased head circumference, neuro-behavioral abnormalities and genitourinary malformations attributable to prenatal exposure to cocaine [9-14].

Political rhetoric has sometimes demonized pregnant crack cocaine users, casting them as immoral and corrupt. Instead of addressing the perceived crack baby epidemic as a public health issue, cocaine-exposed babies were represented as uneducable and worthless drains upon scant public school resources [15]. In turn, the popular media further alarmed the general public with dire predictions for cocaine-exposed infants. However, researchers have countered that there had been a rush to judgment regarding the long-term consequences of prenatal cocaine exposure [16]. Despite such caveats, the criminal justice system responded to a perceived crack baby epidemic by incarcerating pregnant women under drug trafficking laws for delivering illegal substances to minors. Fearing arrest and prosecution may have the undesired consequence of deterring women who may have otherwise sought treatment.

Although prenatal substance abuse extends across class and racial boundaries, the stigma may be identified more with poor and nonwhite women, adding to social inequities [17]. African-American women were reported more likely to lose their cocaine-exposed babies to foster care [18]. Bleak pictures of our social and educational resources being depleted by a generation of crack babies with limited potential have been implied, with investigators cautioning that pediatric psychologists would soon encounter increasing numbers of these children in pediatric clinics, hospitals and schools [19].

However, during the 1990's, as the scientific literature grew, conflicting evidence appeared about cocaine's long-term, non-clinical teratogenic qualities [20]. Coles, et al did not observe negative outcomes with moderate prenatal exposure [21], while replicating earlier findings that cocaine has a negative effect on birth weight and head circumference [22,23]. Some investigators have emphasized that legal substances, e.g., alcohol and nicotine, as well as maternal health and prenatal care, strongly affected reported negative outcomes [24,25]. The importance of determining the maternal age, timing, duration, dose and synergism of polydrug use needs further assessing before drawing conclusions about prenatal exposure to cocaine [26,27]. Crack baby myths must be dispelled before issues such as later reading literacy are addressed [28].

Conspicuously absent in Coles, et al study were extremely adverse neonatal outcomes that were previously reported. Using an improved methodology, Coles, et al found no evidence of urinary-genital tract malformations, no classic withdrawal signs (e.g., tremors, agitation, hyper-tonicity, hyper-reactivity, or gastrointestinal problems) and no significant behavioral aberrations, although some atypical reflex and state regulators were noted. Coles, et al suggested that other drugs in addition to cocaine accounted for significant effects. Incidentally, Coles also reported being vilified as corrupt, inept and advocating drug use for concluding that cocaine's teratogenic potential may have been widely exaggerated.

The widely anticipated, long-term cognitive and social/behavior deficits from intrauterine exposure to cocaine have not been widely observed or reported. It would be premature to unequivocally suggest that prenatal exposure to cocaine produces long-term, global, non-clinical deficits. The extent that public attitudes reflect advances in the science knowledge base or still adhere to the popular stereotypes that began in the 1980's has not been systematically studied.

Establishing more sensible, equitable measures to treat cocaine-addicted mothers and their children is essential for improving U.S. drug policy. Favorable post-natal environments have moderated potential deleterious prenatal effects. However, cocaine is an illicit substance having long been demonized. Since cocaine is illicit and is a relatively well-publicized teratogen, participants may be more likely to impose harsher maternal sanctions in situations involving prenatal exposure to cocaine, than legal drugs also associated with low birth weight and stillbirths. In Study 1 we hypothesized that attitudes toward substance-using pregnant mothers and postnatal outcomes attributable to prenatal cocaine exposure would be more negative than for licit substances. Study 1 examined college student participants' attitudes about criminal sanctions for hypothetical scenarios involving pregnant women's use of one of four substances; cocaine, nicotine, alcohol and caffeine. We hypothesized that in Study 2, positive, negative or neutral media portrayals about long term outcomes were hypothesized to influence viewers' estimations of academic performance and problem behaviors, measured immediately post-viewing. Finding ways of reducing popular crack baby stigmas could influence future policy decisions by legislators.

## Results

### Study 1: Comparing attitudes about prenatal exposure to cocaine, alcohol, nicotine and caffeine

An alpha reliability coefficient of .824 showed high inter-item reliability. A one-way ANOVA (analysis of variance model) showed that a significant difference at .0001 existed between the cocaine, nicotine, alcohol and caffeine conditions when the scores on the items were summed, F(3, 333) = 42.60. The respective means and standard deviations were: cocaine, M (mean) = 5.67, SD (standard deviation) = 1.64; alcohol, M = 4.76, SD = 1.84; nicotine, M = 4.02, SD = 1.8; and caffeine, M = 2.88, SD = 1.79. Post-hoc paired comparisons (Sheffe test, greater than .05 alpha level) showed that cocaine was rated differently from the three other substances.

### Study 2: Comparing effects of positive, neutral or negative media upon immediate post-viewing difference estimates of prenatal cocaine-exposure or non-exposure upon academic performance and risk potential at age 18

A one-way ANOVA for independent samples was performed on the difference scores for the cocaine-exposed and non-exposed teenagers in the hypothetical scenario. There was an overall significant difference between conditions at the.004 level for estimated differences in grade point averages, F (2, 137) = 5.681. Participants in the neutral condition showed a significant disparity for estimated grade point averages for the prenatal cocaine-exposed and non-exposed women at the.0001 apha level, F(1,46) = 86.600. Participants who viewed the negative videotape showed similar results at the .0001 level, F(1, 45) = 127.08. In the negative condition, the estimated cocaine-exposed woman's grade point average was lower than the non-exposed woman's estimated grade point average.

Viewing the positive videotape closed the estimated grade point average difference to a non-statistically significant margin, F(1,45) = 1.263, greater than .05 alpha level. Participants estimated the cocaine-exposed woman's grade point average comparably to the non-exposed woman's grade point average.

Table 2 shows results of estimated risk for problems at age 18 for the prenatal cocaine-exposed and non-exposed women. A statistically significant difference at the .0001 level was obtained for the neutral condition, F(1,46) = 213.14. The estimated risk of problems for the prenatal cocaine-exposed women was greater than for the non-exposed women in the neutral condition.

**Table 2:** Means, standard deviations, difference scores and p values for summed estimated risk of problems (1 – 7 scale) of prenatal cocaine-exposed or non-exposed eighteen year-old women for 3 media conditions.

Media	Exposed	Non-exposed	Difference	p value
Neutral	4.714 (1.258)	2.016 (0.793)	2.698	.0001
Negative	4.528 (1.11)	2.493 (1.012)	2.035	.0001
Positive	3.117 (1.023)	2.768 (0.349)	0.349	> .05

The negative condition also produced a significant difference at the .0001 level for the estimated risk of problems (F(1, 45) = 131.96). The negative presentation produced a larger estimated risk of problems for the prenatal cocaine-exposed woman than the non-exposed one.

Participants who viewed the positive videotape showed no statistically significant differences of estimated risk for problems at the .05 level, F(1,45) = 3.570. The gap between the estimated problems at age 18 for the cocaine-exposed and non-exposed women was reduced in the positive media condition.

## Discussion

In all conditions, the prenatal cocaine-exposed teen was rated as having lower academic achievement and having more risk of problems. These results show there is a strong societal expectation that prenatal cocaine exposure will produce long-term academic deficits and other problems.

Compared to participants in the other two conditions, participants viewing the positive presentation slightly increased grade point average ratings for the prenatal cocaine-exposed teen. However, participants in the positive media condition also reduced their grade point average ratings for the non-exposed teen. The same effect occurred for estimated problems. These findings suggest that participants were conflicted by viewing the positive media portrayal. Compared to participants in the neutral and negative conditions, participants in the positive condition reduced cognitive dissonance by simultaneously enhancing ratings of the prenatal cocaine-exposed woman and diminishing their ratings for the non-exposed one.

## Conclusion

Nurturing postnatal environments may help reduce deficits produced from prenatal problems, a finding that has drawn relatively little public attention. Lester and Tronick [29] offered several examples, e.g., substance abuse, pre-term delivery, low birth weight. Since all of the mothers in their sample had continued in drug treatment program, Lester and Tronick speculated that this intervention could have mitigated prenatal exposure to crack cocaine and other substances. They emphasized that when infants who were exposed to a wide variety of potentially harmful prenatal insults develop in a reasonably sound home environment, they should be expected to do almost as well as any infant. In contrast, a hostile postnatal environment can aggravate earlier negative prenatal influences and jeopardize the potential for normal patterns of growth and development. They further suggested that responsive postnatal environments noted in their investigation were not considered in past research and may have been one reason that previous investigators had failed to find significant outcome differences in a previous three-year follow-up of their neonatal sample [30].

The findings of Study 1 showed that the sample held different attitudes about possible criminal sanctions and developmental problems for cocaine, compared to attitudes about alcohol, nicotine and caffeine. Results from Study 2 showed that stereotyped attitudes were modified, at least in the short-term, by media portrayals. However, the cognitive dissonance effect observed in the positive media condition raises doubts about whether these relatively more positive attitudes observed in the short-term would persist. Our findings did not address long-term attitude changes, or whether the data may generalize to a broader population sample. Finding mechanisms for reducing popular crack baby stigmas in the long-term may essentially influence future policy decisions by legislators. Establishing more sensible, equitable measures to treat cocaine-addicted mothers and their children is essential for improving U.S. drug policy. Favorable post-natal environments have moderated potential deleterious prenatal effects, a hopeful finding that should be emphasized in the continuing policy dialogues and media characterizations about cocaine's harmful effects and the treatment of affected women and children. Reducing popular crack baby stigmas could influence future policy decisions by legislators.

## Methods

### Study 1: Comparing attitudes about prenatal exposure to cocaine, alcohol, nicotine and caffeine

We compared 336 university students' evaluations of criminal justice sanctions against mothers who potentially harmed their fetuses through prenatal ingestion of one of four substances associated with low birth weight and possible stillbirths: cocaine, alcohol, nicotine and caffeine.

The participants were 336 undergraduate students enrolled in Introduction to Psychology (M = 19.8 years, SD = 4.52). The sample was comprised of 222 females and 114 males. Ethnic composition of the sample was; White (69%), Hispanic (16%), African-American (9%), Asian-American (2%), Native-American (1%), and Other (3%).

Participants read a printed hypothetical scenario about a pregnant woman who tested positive for one of the substances. The opening paragraph of the hypothetical situation described the pregnant woman's use of one substance; cocaine, nicotine, alcohol or caffeine. The woman, Ms. Johnson, tested positive for the substance while she was pregnant. She refused to participate in free mandatory counseling. She faced prosecution in her state for child abuse. A series of questions about criminal sanctions followed the scenario. Responses were recorded on a 1-to-7 scale following each question.

**Figure 1:**
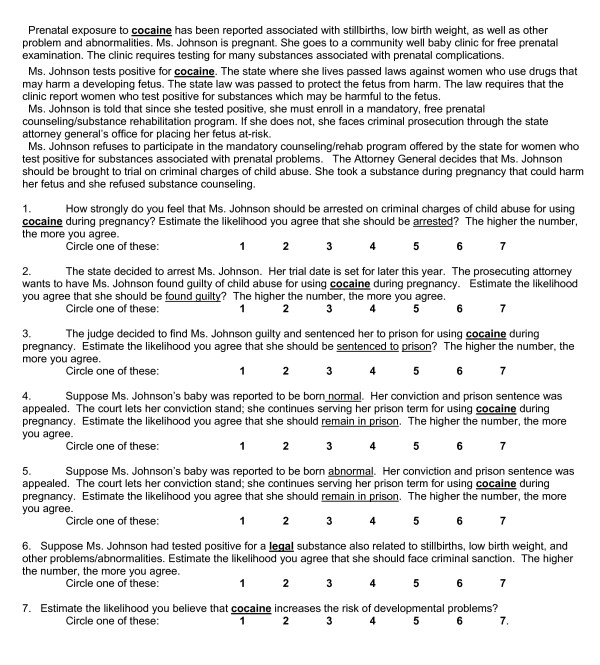
Scenarios and ratings for criminal sanctions and developmental risks for cocaine nicotine, alcohol or caffeine.

Participants were also asked to estimate the likelihood that the particular substance in their hypothetical scenario increased the risk of developmental problems. Figure 1 shows the survey instrument for one of the four substances, cocaine. Alcohol, nicotine and caffeine were substituted in the parallel forms.

The numbers of participants randomly assigned to conditions where the substance described in the scenario for cocaine, nicotine, alcohol or caffeine were 84, 82, 79 and 81, respectively. The only difference between the conditions was the designated substance. Participants were asked to read the scenarios and rated the extent to which they agreed or disagreed with the statements about sanctions and risks for that substance.

### Study 2: Comparing effects of positive, neutral or negative media upon immediate post-viewing difference estimates of prenatal cocaine-exposure or non-exposure upon academic performance and risk potential at age 18

We examined whether media portrayals could influence attitudes about long-term effects of prenatal cocaine exposure. After participants viewed positive, negative or neutral videotapes, we compared their ratings of the estimated grade point averages and risks of problems for cocaine-exposed and non-exposed eighteen year-old women.

Participants were 145 undergraduate students enrolled in psychology courses. Data was collected from 139 participants who completed the response sheets (M = 21.3 years, SD = 4.08 years). The sample was comprised of 100 females and 39 males.

**Figure 2:**
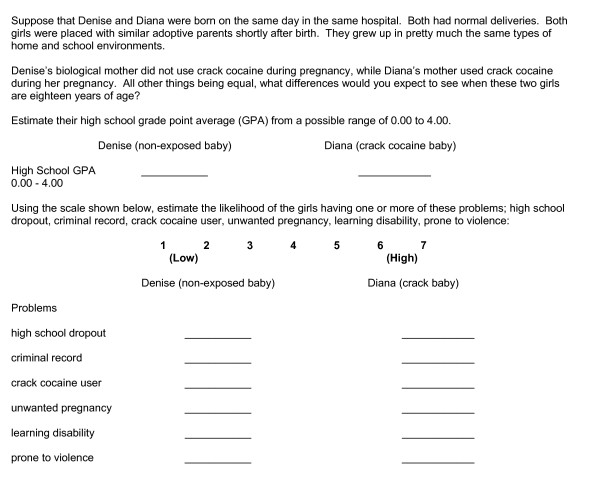
Scenarios for prenatal cocaine-exposed and non-exposed eighteen year-old women with ratings for estimated grade point averages and estimated risks for problems.

Printed hypothetical scenarios about two adopted female babies were presented to participants. One was cocaine-exposed during pregnancy, while the other was not. The girls grew up in equivalent, caring adoptive homes. Participants estimated the grade point average and risks of one or more problems (e.g., criminal record, crack cocaine use, unwanted pregnancy, high school dropout, learning disability, prone to violence) at age eighteen for the two women. Figure 2 shows the scenario and participants' response sheet.

Three 12-minute videotape presentations were used. The neutral media condition was about space exploration. The negative media condition showed how teratogens enter the intrauterine environment and showed a neonate exhibiting hyper-tonicity, tremors and irritability attributed to prenatal cocaine exposure. The positive videotape was an elaboration of how preliminary research on prenatal cocaine exposure was methodologically flawed. The positive media condition featured an 18 year-old woman whose mother had used crack cocaine during pregnancy. She had been adopted by a caring family and showed no deleterious effects of prenatal exposure to crack cocaine. In her testimonial, the teen described how she had been negatively stereotyped as a crack baby. The presentation illustrated how this crack baby was an apparently normal, well-adapted and thriving adolescent.

The positive videotape concluded with the narrator, ABC television news journalist John Stossel commenting about the importance of falsifiable hypotheses in science and how the scientific process is self-correcting. He noted that future research might contradict earlier research. The closing narrative stated that information represented on the program might also be revised through the slow and often arduous scientific process.

There were 46 participants assigned at random to the positive, 46 assigned to negative and 47 assigned to neutral conditions. They entered the viewing room and watched the respective program as a group. After viewing, participants read the scenario about two adopted neonates, Denise and Diana. All other things being equal in their lives, one had prenatal exposure to cocaine while the other infant was not exposed to prenatal cocaine. Participants completed their responses to questions about estimated grade point averages and estimated risk of problems at age 18 for the cocaine-exposed and non-exposed infants.

## Competing interests

The author(s) declare that they have no competing interests.

## Authors' contributions

HJG drafted and revised the manuscript and was responsible for concept and design.

PR performed analyses and was responsible for interpretation of results.

KLA also performed statistical analyses and was responsible for data interpretation.

ASB was also responsible for concept and design.

**Table 1:** Means, standard deviations, difference scores and p values for estimated grade point averages (0.00 to 4.00) of prenatal cocaine-exposed or non-exposed eighteen year-old women for 3 media conditions.

Media	Exposed	Non-exposed	Difference	p value
Neutral	2.333 (0.568)	3.123 (0.262)	0.791	.0001
Negative	2.335 (0.582)	3.253 (0.918)	0.918	.0001
Positive	2.933 (0.443)	3.029 (0.358)	0.096	> .05
